# Integration of a collaborative robotic platform in laparoscopic cholecystectomy: safety, feasibility, and operative time variability

**DOI:** 10.1007/s11701-026-03478-3

**Published:** 2026-05-23

**Authors:** Azagury Dan, Mercoli Henry, Leclerc Fabrice, Reitano Elisa

**Affiliations:** 1 Minimally Invasive & Bariatric Surgery, Stanford University School of Medicine, Stanford, CA USA; 2 Digestive Surgery, Polyclinique de Franche-Comté ELSAN, 4 rue Rodin, Besançon, 25000 France; 3 Digestive Surgery, IHU-mix Surg, Hopitaux Universitaires de Strasbourg, Strasbourg, France

**Keywords:** Cholecystectomy, Robotic surgery, Maestro assistance, Artificial intelligence

## Abstract

Laparoscopic cholecystectomy is a high-volume procedure with relatively short operative times, leaving limited margin for further reduction in mean duration. However, variability in operative performance, compounded by anatomical variation and differing degrees of inflammation, may substantially affect operative duration and operating room efficiency. The Maestro™ Platform is a bedside, AI-powered, co-manipulated surgical assistance system designed to improve visualization, stability, and ergonomics during MIS while preserving surgeon autonomy, standard laparoscopic instrumentation, and conventional operative technique. This study aims to assess the operative and perioperative outcomes of Maestro-assisted versus conventional laparoscopic cholecystectomy, with a focus on variability in operative time. This study prospectively included 79 Maestro-assisted cholecystectomies procedures. Those cases were retrospectively compared with 79 consecutive baseline laparoscopic cholecystectomies performed by the same surgical team. Demographic data, operative time, length of stay and 30-day complications were analyzed. Statistical Process Control (SPC) charts were generated to evaluate the process stability and variability for operative times. The Maestro demonstrated a 35% reduction in operative time variability (*p* < 0.001). The SPC charts show significantly reduced variability and greater process stability with the Maestro Platform than conventional laparoscopy. Postoperative outcomes were comparable. No device-related complications or conversions occurred. Maestro-assisted cholecystectomy demonstrated operative time and perioperative outcomes comparable to conventional laparoscopy, while showing a significant reduction in operative time variability. For short procedures, this improved predictability may offer substantial workflow and scheduling advantages, such as more on-time starts and reduced overtime hours.

## Introduction

Gallstone disease is one of the most common digestive disorders worldwide, and laparoscopic cholecystectomy has supplanted open surgery as the standard treatment [1]. Despite the procedure’s ubiquity, conventional laparoscopy relies heavily on human assistants - with variable levels of experience - for camera endoscope positioning and liver and tissue retraction, introducing variability in visualization, exposure, and workflow. Inconsistent camera control and delays in adjustment and cleaning, as well as ergonomic strain for both surgeons and assistants, can negatively affect operative efficiency and procedural consistency. As a result, the quality of endoscopic control, tissue retraction, and intraoperative flow remains a critical determinant of surgical performance and outcomes.

Tele-robotic surgical platforms were developed to address these limitations by transferring control of visualization and retraction directly to the surgeon. While this approach can reduce assistant-related variability, it requires the surgeon to operate from a remote console outside the sterile field. In routine procedures such as cholecystectomy, tele-robotic systems have been associated with longer operative times, and, in some series, higher rates of bile-duct injury, without consistent improvements in length of stay or outcomes [2–4]. These tradeoffs have limited their utility in high-volume, efficiency-focused settings.

As a result, there is a growing need for innovative solutions that bridge the gap, the combines the valuable aspects of both the robotic and laparoscopic approaches into a single platform without the infrastructure demands associated with the current robotic platforms.

Collaborative surgical platforms are bedside robotic systems designed to provide the surgeon with direct control of visualization and instrument retraction during minimally invasive procedures. By enabling stable and responsive control of the endoscope and selected instruments under the surgeon’s command, these systems reduce reliance on human assistants while preserving tactile feedback, situational awareness, and integration within the standard laparoscopic workflow.

Unlike console-based tele-robotic platforms, collaborative systems are operated entirely at the patient’s bedside and do not require surgeon relocation outside the sterile field. This configuration allows immediate adjustment of exposure and visualization while maintaining team coordination and operative continuity.

The Maestro™ Surgical System (Moon Surgical, Paris, France) is a collaborative robotic platform developed to support conventional laparoscopic instruments and laparoscopes. Rather than replicating the surgeon’s hand movements from a remote console, the system provides powered, stable instrument holding and camera control integrated into the existing laparoscopic setup. In contrast to conventional large robotic systems that place the surgeon away from the patient and rely on additional bedside personnel, Maestro maintains a fully bedside surgical workflow [5, 6]. Early experience in laparoscopic general surgery suggests that such collaborative robotic platforms are associated with shorter learning curves and improved operative efficiency compared with larger, cost-intensive robotic systems [7, 8]. However, despite increasing adoption, comparative data evaluating their impact on procedural performance in cholecystectomy remain scarce. The present study aims to compare operative performance and variability between Maestro-assisted and conventional laparoscopic cholecystectomy. In this context, collaborative platforms aim not to reduce already optimized mean operative times, but to improve procedural consistency by reducing variability related to assistant-dependent factors. Evaluated outcomes include operative duration, operating room time, variability in operative time, device-related complications, and overall complications.

## Materials and methods

This was a single-centre retrospective comparative cohort study conducted at Polyclinique de Franche-Comté (Elsan, Besançon, France). All procedures were performed by two senior experienced laparoscopic surgeons using standardized surgical techniques. The study compared outcomes before and after implementation of the Maestro collaborative robotic platform.

All procedures were performed electively. 79 Maestro-assisted cholecystectomies procedures were included prospectively between October 2023 and September 2024.

Those were compared with the 79 consecutive standard laparoscopic cholecystectomies performed immediately prior to Maestro implementation. To minimize operator-related confounding, surgeon identity was required to be identical across cohorts, and all procedures were performed within the same institutional setting and perioperative care pathway. Baseline patient characteristics, including age, BMI, and ASA physical status, were comparable between groups.

Eligible patients were adults over 18 undergoing elective or urgent laparoscopic cholecystectomy for symptomatic gallstone disease or chronic and acute cholecystitis. Exclusion criteria included planned open surgery or concomitant major procedures.

Statistical Process Control (SPC) charts provide a visual way to track how operative performance evolves over consecutive cases. By separating normal random variation from meaningful process changes, SPC analysis helps determine whether a surgical workflow is stable and consistent, or if special factors are introducing variability.

To evaluate temporal stability and process consistency, control charts (SPC I-charts) were constructed for the operative time (skin-to-skin (S2S) time) in both cohorts.

Cases were plotted in chronological order according to the operation date, as this sequence best reflects the real-time evolution of the surgical process and allows assessment of both process control and potential learning-curve effects.

For each group, the process mean and the upper and lower control limits (UCL/LCL) were defined as the mean ± 3 standard deviations.

Additional process indicators, including the standard deviation (SD), coefficient of variation (CV%), and number of out-of-control points, were used to quantify process variability and stability.

This investigation was conducted within the framework of a post‑market clinical registry evaluating Maestro at one center. The registry was duly declared to the French Data Protection Authority (CNIL, reference n°223283).

### The Maestro™ Platform

The Maestro™ Platform (Moon Surgical, Paris, France) is a two-arm surgical platform designed to support visualization and tissue exposure during minimally invasive surgery under the surgeon’s direct control. Unlike console-based robotic systems that separate the surgeon from the patient and rely on remote manipulation, Maestro is controlled directly by the surgeon at the bedside, allowing continuous interaction with the operative field.

**Fig. 1:**
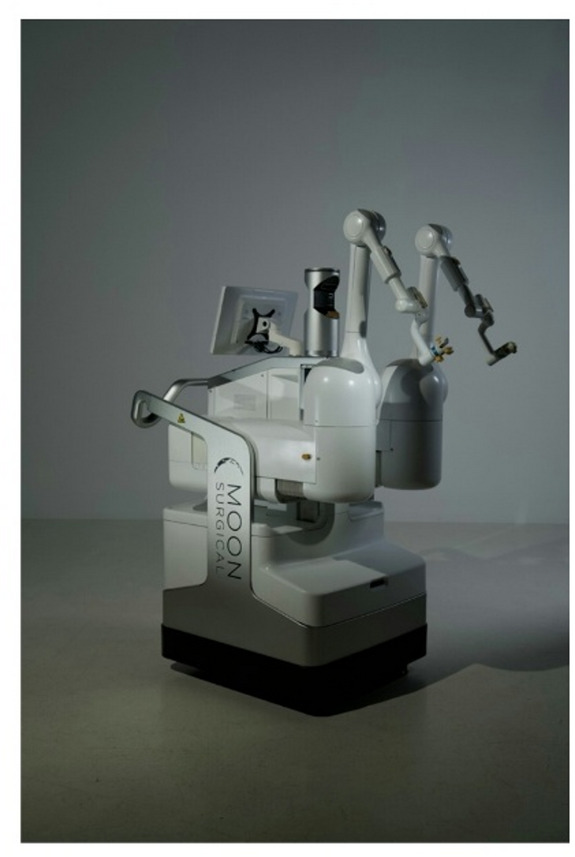
Maestro™ Platform

The system features two articulated robotic arms mounted on a mobile cart positioned alongside the operating table (Fig. 1). These arms accommodate standard 5-mm and 10-mm laparoscopic instruments, including laparoscopes and graspers. The surgeon performs all dissections manually, while the platform provides camera support and assists with tissue retraction. One arm controls the laparoscope, while the other supports tissue retraction and exposure.

A central component of the technology is the ScoPilot™ module, an integrated computer-vision system that detects the movement of operative instruments and can automatically adjust the laparoscope to maintain appropriate framing of the surgical field. This optional feature may reduce the need for repeated manual camera adjustments.

**Fig. 2:**
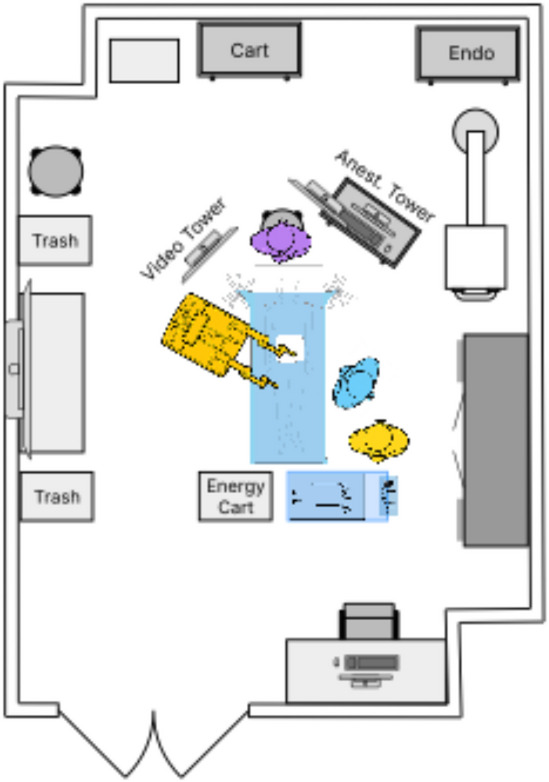
Schematic operating-room layout for laparoscopic cholecystectomy with Maestro. (Maestro shown in yellow)

Maestro integrates with existing laparoscopic equipment and does not require specialized instruments or trocars. Because the surgeon remains scrubbed and at the patient’s side throughout the procedure, the platform preserves the standard laparoscopic workflow while reducing reliance on assistant-dependent camera control and retraction. Its compact footprint (9 square ft) and compatibility with standard instruments are intended to support procedural consistency without fundamentally altering established operative techniques (Fig. 2).

### Surgical technique

Both groups underwent a standard four-trocar laparoscopic cholecystectomy with systematic identification of the critical view of safety. 1 × 10 mm trocar for optical port and 3 5 mm trocars for retraction and dissection. Intra Operative Cholagiogram (IOC) was not systematically used based on patients’ anatomies, condition and surgeon preference. Dissection was performed using off the shelf monopolar hook and bipolar cautery when needed.

Once the critical view of safety was obtained, the cystic duct and artery were clipped and divided, and the gallbladder removed from the liver and extracted through a bag for pathological examination.

**Fig. 3:**
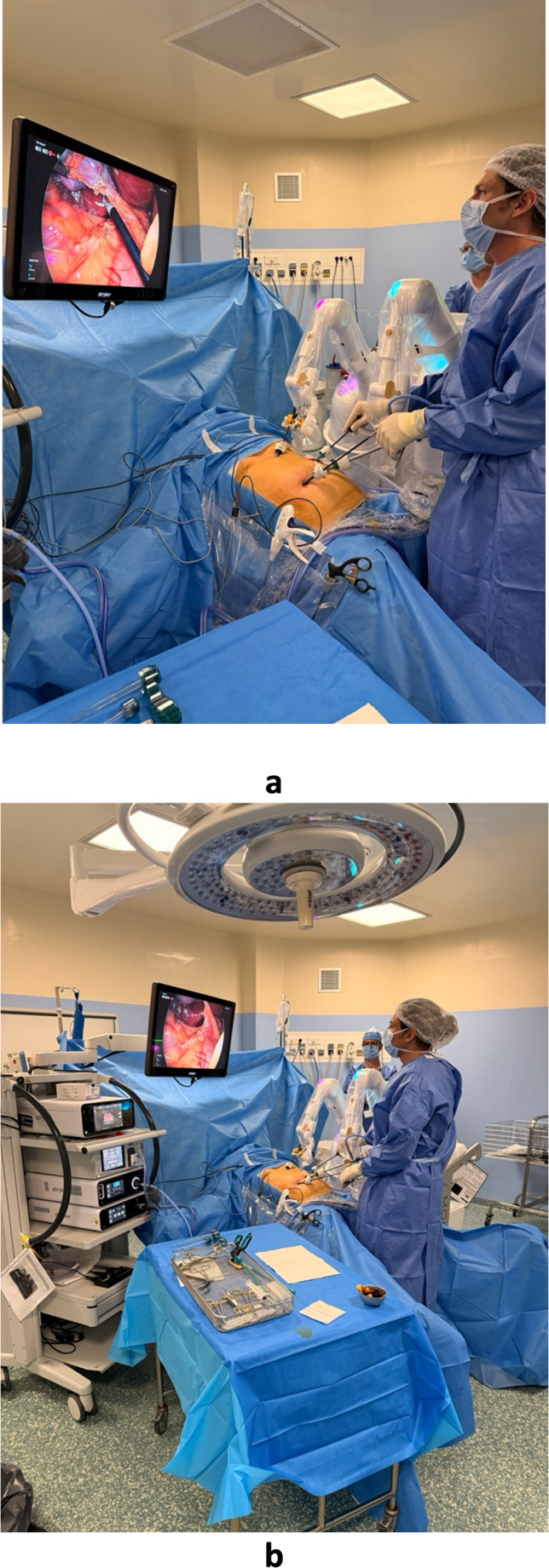
Operative setup in Maestro-assisted procedure. **(a)** Operative setup and visualization conditions in Maestro-assisted procedure (close-up view). **(b)** Operative setup and visualization conditions in Maestro-assisted procedure (wide-angle view)

**Fig. 4:**
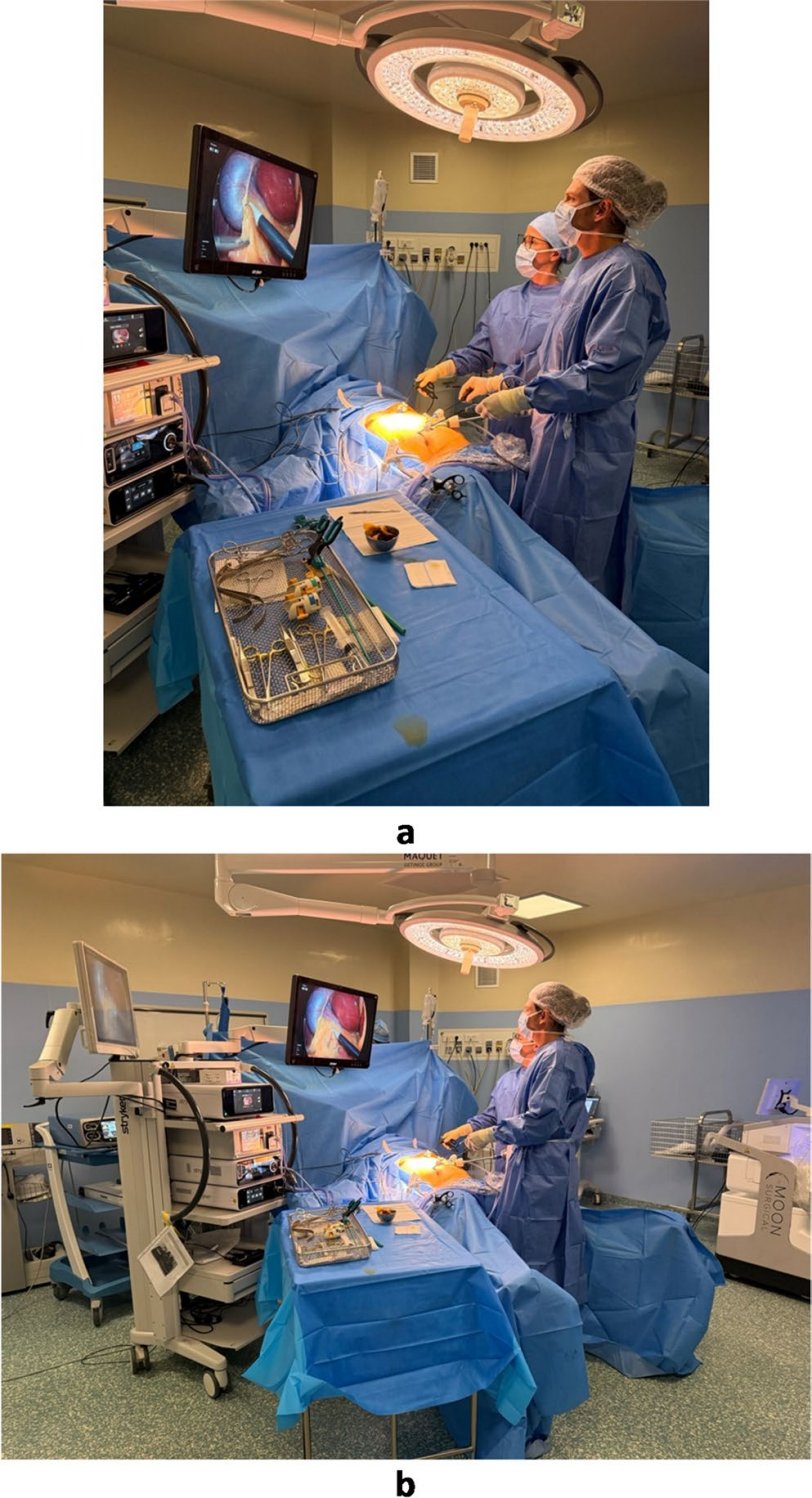
Operative setup in standard laparoscopic procedure. **(a)** Operative setup and visualization conditions in standard laparoscopic procedure (close-up view). **(b)** Operative setup and visualization conditions in standard laparoscopic procedure (wide-angle view)

In the Maestro cohort, the laparoscope and a retractor instrument were mounted on the two articulated robotic arms, while the surgeon performed all dissection manually at the bedside (Fig. 3a and b). The conventional cohort used standard assistant-held instruments (Fig. 4a and b).

### Data collection and outcomes

For each patient, demographic variables (age, sex, ASA, BMI), incision-to-closure time, length of stay (LOS), 30-day postoperative complications and unplanned readmissions were collected.

### Statistical analysis

Continuous variables are reported as mean ± SD or median (IQR). Means were compared with Welch’s t-test. Operative time variability was evaluated using SD, CV, and an F-test for variance. Categorical variables were compared using chi-square or Fisher’s exact test. A two-sided *p* < 0.05 was considered statistically significant.

## Results

### Patients characteristics

A total of 158 patients were analyzed (79 per cohort). Baseline characteristics were comparable, with no clinically relevant differences in age, sex distribution or ASA. A higher mean BMI was observed in the conventional cohort compared to the Maestro cohort (28.0 vs. 26.1 kg/m², *p* = 0.0014), although both groups remained within comparable clinical ranges.

The patients analyzed in the Maestro™-enhanced group had a mean age of 56.5 (SD 17.43) years and a mean BMI of 26.1 (SD 4.3) kg/m^2,^ and a mean ASA of 2.2 (SD 0.63). There were 56 women (71%) and 23 men (29%).

The patients in the conventional laparoscopic cholecystectomy group had a mean age of 58.3 (SD 16.86) years, a mean BMI of 28.0 (SD 5.26), and a mean ASA score of 2.2 (SD 0.7). There were 59 women (75%) and 20 men (25%).

The two surgeons each contributed a balanced number of cases to both groups. Full details of the patient demographics are shown in Table 1.

**Table 1:** Patients Characteristics

Values	Maestro- assisted cholecystecomies	Standard Laparoscopic cholecystecomies	*p*-value
Gender- Female (n, %)	56 (71%)	59 (75%)	0.59
Average of Age at Time of Surgery, years (SD)	56.5(17.4)	58.3 (16.9)	0.52
Median ASA Classification (Range)	2.2 (1–3)	2.2 (1–3)	0.84
Mean Body Mass Index, kg/m2 (SD)	26.1 (4.3)	28.0 (5.3)	0.0014

### Operative performance

Mean operative time was nearly identical between groups (28.96 ± 10.06 min with Maestro vs. 29.01 ± 15.55 min with conventional laparoscopy; *p* = 0.98).

In addition, the use of the Maestro™ platform was associated with a significant 35% reduction in operative time variability : Maestro™-enhanced cholecystectomy : SD 10.1 vs. laparoscopic cholecystectomy : SD 15.5 (*p* < 0.001).

### Length of stay and postoperative outcomes

Mean LOS as well as day-case discharge rates were comparable (0.24 vs. 0.19 days; *p* = 0.63, 84.8% of Maestro cases and 88.6% of standard laparoscopic cases; *p* = 0.64).

Postoperative complications rates (2.5%) and severity from both cohorts were comparable, and 30-day readmission rates were identical (2/79; 2.5% each). No reoperations or mortality were recorded.

In the baseline group, one patient developed a postoperative biliary collection requiring CT-guided percutaneous drainage (Clavien–Dindo grade IIIa), and one patient experienced minor bleeding at the umbilical wound, managed with bedside measures.

In the Maestro group, one patient presented with a superficial infection of the umbilical wound treated with antibiotics, and one patient reported postoperative abdominal pain requiring analgesics, which resolved spontaneously.

There were no device-related complications or conversions in the Maestro-assisted group.

Full details of intraoperative and postoperative outcomes are shown in Table 2.

**Table 2:** Intraoperative and Postoperative Outcomes

Values	Maestro- assisted cholecystecomies	Standard Laparoscopic cholecystecomies	*p*-value
Mean Duration of Surgical Procedure, min (SD)	29.0 (10.1)	29.0 (15.5)	> 0.05 (0.98)
Operative Time Variability, SD	10.1	15.5	< 0.05 (0.0002)
Intraoperative Conversion (n, %)	0 (0%)	0 (0%)	
Post-operative Complications (n, %)	2 (2.5%)	2 (2.5%)	
Umbilical wound infection (n, %)	1 (1.3%)	0	NA
Abdominal Pain (n, %)	1 (1.3%)	0	NA
Biliary collection (n,%)	0	1 (1.3%)	NA
Umbilical wound bleeding(n,%)	0	1 (1.3%)	NA
Device related adverse events	0	NA	NA
Length of Stay, mean (days)	0.24	0.19	> 0.005 (0.63)
Day-case discharge, n (%)	67 (84.8%)	70 (88.6%)	> 0.005 (0.64)

### Process variability and stability

Statistical Process Control (SPC) charts provide a visual representation of how operating times evolve over time, allowing both variability and process stability to be assessed. Each point on the chart represents an individual case performed sequentially, while the central line represents the overall mean operative time. The upper and lower control limits (UCL and LCL) delineate the expected range of normal variation, typically set at ± 3 standard deviations from the mean. When all data points remain within these limits and show no systematic trends, the process is considered “in control,” meaning that the observed variations are random and not due to identifiable external causes.

Statistical process control charts were generated for the 79 cases in each cohort, using chronological case order to visualize the evolution of operative times. These charts allowed assessment of both process variability and stability.

**Fig. 5:**
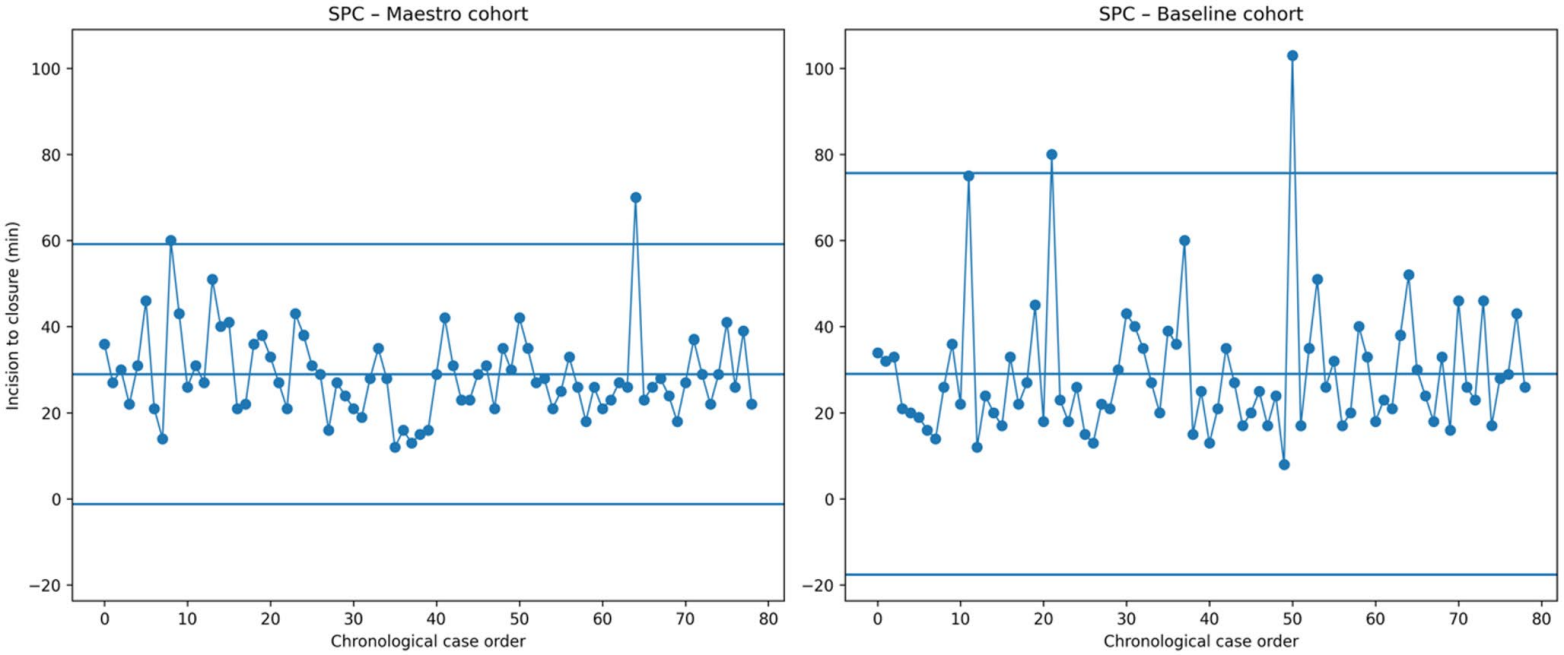
SPC Charts for operative time – Maestro™ vs. Baseline. Statistical Process Control (SPC) charts display operating time sequentially over time, with a central mean and upper and lower control limits set at ± 3 standard deviations. Points outside these limits indicate special-cause variation and a process that is statistically out of control

In the present study, the SPC charts demonstrated that the Maestro system yielded a narrower range of operative time variation (UCL 59 min, LCL − 1 min) compared with conventional laparoscopy (UCL 76 min, LCL − 18 min) (Fig. 5). Statistical process control analysis revealed clear differences in the dispersion of operative times between the two cohorts. In the Maestro-assisted group, operative times were more tightly clustered around the mean, resulting in narrower upper and lower control limits compared with the baseline cohort (Fig. 5). This pattern reflects a substantial reduction in process variability and greater procedural consistency over time. By contrast, the baseline cohort demonstrated wider control limits and more pronounced fluctuations between consecutive cases, driven by several prolonged procedures that increased overall dispersion.

The Maestro SPC chart further displayed a smoother temporal profile with fewer abrupt case-to-case deviations, consistent with a more standardized and predictable surgical workflow.

## Discussion

In this comparative study, Maestro-assisted cholecystectomy achieved near identical operative times and perioperative outcomes comparable to conventional laparoscopy, confirming that a collaborative robotic platform can be integrated into a highly standardized procedure while preserving workflow efficiency and patient safety. Given the short duration and reproducible workflow of laparoscopic cholecystectomy, the absence of a reduction in mean operative time was expected.

The most relevant finding of this study is the reduction in operative time variability observed with the use of Maestro. While average operative duration was comparable across groups, Maestro significantly reduced the dispersion of operative times, resulting in a more predictable surgical process. For high-volume procedures such as cholecystectomy, variability rather than mean duration is often the primary constraint on operating room performance. Improved predictability may facilitate case scheduling, reduces overruns, and supports more efficient utilization of operating room resources, particularly in mixed elective programs.

These results reflect the underlying design philosophy of collaborative robotics. By providing stable camera control and consistent dynamic retraction while preserving a fully unconstrained bedside workflow, Maestro reduced the variability introduced by conventional assistance and limits workflow disruptions. Unlike console-based robotic systems, which physically separate the surgeon from the patient and introduce additional layers of complexity, Maestro allows surgeons to remain scrubbed at the bedside, maintain haptic feedback, and operate using standard laparoscopic instruments. In this context, the observed reduction in variability likely represents improved consistency of exposure and workflow rather than changes in surgical technique.

Conventional laparoscopic cholecystectomy remains the reference standard due to its efficiency, low cost, and well-established safety profile. Its reproducibility makes it particularly suitable for high-volume settings. However, it relies on human assistance for camera control and retraction, which may introduce variability in visualization and intraoperative workflow.

Robotic-assisted platforms offer improved surgeon autonomy, stable visualization, and enhanced ergonomics, potentially reducing assistant dependency. However, these advantages must be balanced against increased capital and operational costs, longer setup times, and, in some reports, longer operative durations. These constraints raise questions regarding their value in short procedures where mean operative time is already optimized.

In this context, collaborative robotic platforms represent an intermediate approach, aiming to combine the advantages of both techniques. By providing consistent visualization and retraction under the surgeon’s direct control while preserving a bedside workflow and the use of standard laparoscopic instruments, such systems seek to reduce variability without compromising procedural efficiency.

Building on these considerations, our findings contribute to the broader discussion surrounding the role of robotics in cholecystectomy. Prior studies evaluating tele-robotic platforms have reported mixed results, including longer operative times and, in some series, higher complication rates compared with conventional laparoscopy, especially regarding common bile duct injuries [3]. These systems offer technical precision but require substantial capital investment, increased staffing, and longer setup times. Collaborative robotic platforms such as Maestro occupy a distinct middle ground, aiming to deliver selected benefits of robotics—stability, autonomy, and reproducibility—without the cost, complexity, or workflow disruption associated with console-based systems.

For surgeons, the platform offers greater autonomy over visualization and exposure while reducing physical strain and dependence on assistant availability. For hospitals and ambulatory centers, the small footprint, rapid setup, and staffing efficiencies associated with collaborative robotics may support higher throughput and more predictable operating room utilization. These attributes are particularly relevant as health systems increasingly shift routine laparoscopic procedures toward outpatient and short-stay settings.

This study has limitations, including its single-center design and the absence of randomization. Although randomization was not feasible in this early clinical evaluation, the use of consecutive cases immediately preceding implementation, combined with surgeon-level matching and comparable baseline characteristics, was intended to limit selection and temporal biases. In addition, no formal grading of operative difficulty (for example based on inflammation severity or intraoperative complexity scoring) was performed. While the conventional group had a 2 points higher mean BMI than the Maestro group, this difference did not translate into differences in mean operative time or postoperative outcomes. The primary finding of the study relates to operative time variability, which remained significantly different between groups despite similar mean operative times Nevertheless, in line with the IDEAL framework, these findings represent an important exploration in the evaluation of collaborative robotics. Further prospective, multicenter studies are warranted to assess economic impact, surgeon ergonomics, learning curves, and broader safety outcomes, including adherence to safe cholecystectomy principles.

## Conclusion

In this comparative study, Maestro-assisted cholecystectomy demonstrated operative time and perioperative outcomes comparable to conventional laparoscopy, suggesting that integration of a collaborative robotic platform into routine practice is feasible and safe.

The use of Maestro was associated with a significant reduction in operative time variability, reflecting a more consistent and predictable surgical procedure, with no changes in mean operative time.

In high-volume surgical settings, where variability rather than mean duration often drives inefficiency, this improvement in procedural consistency may have meaningful implications for operating room management, including more reliable scheduling and reduced risk of overruns. These findings suggest that collaborative robotic platforms may offer a pragmatic and scalable approach to enhancing surgical workflow, not by accelerating procedures, but by improving their reproducibility and predictability.

## Data Availability

All data supporting the findings of this study are available within the paper and tables.
